# Treatment of SLAP Lesions

**DOI:** 10.2174/1874325001812010288

**Published:** 2018-07-31

**Authors:** Apostolos Stathellis, Emmanouil Brilakis, Jim-Dimitris Georgoulis, Emmanouil Antonogiannakis, Anastasios Georgoulis

**Affiliations:** 1 3^rd^ Orthopaedic Department, Hygeia Hospital, Athens, Greece; 2 University of Ioannina, Orthopaedic Department, Ioannina, Greece; 3 Department of General Surgery, General Hospital of Corfu, Greece

**Keywords:** SLAP lesion, Shoulder, Biceps tendon, Tenotomy, Tenodesis, Treatment algorithm

## Abstract

**Background:**

The surgical treatment of a Superior Labrum Anterior and Posterior (SLAP) lesion becomes more and more frequent as the surgical techniques, the implants and the postoperative rehabilitation of the patient are improved and provide in most cases an excellent outcome.

**Objective:**

However, a standard therapy of SLAP lesions in the shoulder surgery has not been established yet. An algorithm on how to treat SLAP lesions according to their type and data on the factors that influence the surgical outcome is essential for the everyday clinical practice.

**Method:**

In this article, a retrospective evaluation of patients with SLAP lesion, treated surgically in our orthopaedic clinic was conducted.

**Results:**

According to the clinical outcome and our experience with the surgical therapy of SLAP lesions we demonstrate an algorithm on the proper therapeutic approach.

**Conclusion:**

SLAP I lesions are treated with debridement. Most controversies concern patients with SLAP II lesions, whose therapy is either fixation of the superior labrum or tenotomy/tenodesis of the long head of the biceps tendon. For patients with SLAP III or IV lesions the most commonly accepted approach is tenotomy or tenodesis of the long head of biceps tendon.

## 1 INTRODUCTION

Andrews *et al*. described in 1985 initially the Superior Labrum Anterior and Posterior (SLAP) lesion [1, 2]. Snyder first presented in 1990 a comprehensive classification of the SLAP lesions [3]. The SLAP lesions were with this classification separated in four different types. The Snyder classification provides an anatomic description of the SLAP lesion as well as a general picture of the severity of the injury. The diagnosis of the lesion is often made preoperatively *via* MRI imaging. However, the exact classification of the SLAP lesion should be made intraoperatively during the shoulder arthroscopy.

## 2 SNYDER CLASSIFICATION

### 2.1 Type I

These lesions are characterized by degeneration of the superior labrum free edge with an intact peripheral attachment and stable anchor of the long biceps tendon.

### 2.2 Type II

These lesions are described as a detachment of the superior labrum and biceps from the glenoid with an unstable biceps anchor.

### 2.3 Type III

The main characteristic of these lesions is a bucket-handle tear of the superior labrum with an intact biceps tendon anchor.

### 2.4 Type IV

In this type of lesion a bucket-handle tear of the superior labrum is identified, which is displaced in the joint together with the biceps tendon anchor.

SLAP lesions can lead to shoulder pain, mechanical symptoms and impaired function of the joint. They are often associated with other shoulder pathologies, such as Bankart lesion or rotator cuff tears. It is necessary to separate a SLAP lesion from other normal anatomic variations of the shoulder, such as the Buford complex or a sub-labral hole. The Buford complex is characterized from a thick cord-like middle glenohumeral ligament and the absence of anterosuperior labral tissue. The sub-labral hole is described as a groove between the normal anterosuperior labrum and the anterior cartilaginous border of the glenoid rim [4, 5]. The function of the long head of the biceps tendon is controversial. Some authors argue that the tendon has no role in glenohumeral stability or humeral migration. Other surgeons believe that the biceps tendon has a role as a secondary glenohumeral stabilizer [6]. A SLAP lesion can be the result of a variety of injury mechanisms [2], in most cases overuse injuries. In overhead athletes, the repeated throwing motion of the shoulder can cause micro-injury to the superior labrum. Initially a posterosuperior labrum tear develops, which can be gradually extended forward to the biceps anchor. Another injury mechanism for a SLAP lesion is a fall on the outstretched arm [2].

The pain pattern is non-specific. Most of the patients complain about a dull pain in the shoulder joint. Diagnosis of a SLAP lesion based only on the clinical examination is usually very difficult with most of the clinical tests being non-specific. One reason is that the detached labrum rarely causes mechanical symptoms, such as blocking of the joint. Another reason is that most patients have also coexisting pathologies in the shoulder, which cause similar symptoms and complicate the physical examination [7]. Preoperative imaging is very important for the diagnosis of SLAP lesion. Magnetic Resonance Arthrography (MRA) is the gold standard. The contrast medium distends the joint capsule, outlines the intraarticular structures revealing the detachment of the superior labrum, which is best identified in the coronal images. The sensitivity of MRA is reported in several studies to reach up to 90%. With the MRA spinoglenoid cysts can also be diagnosed. These cysts can cause entrapment of the suprascapular nerve, leading to dull shoulder pain to the patient, similar to the pain caused by a SLAP lesion [4, 8].

## 3 MATERIAL AND METHODS

We performed a retrospective evaluation of our patient collective with SLAP lesion. According to the type of the lesion, age and activity level of the patient, we performed an individualized surgical treatment. The different surgical options for the treatment of a SLAP lesion are: i) debridement of the superior labrum ii) fixation of the superior labrum to the glenoid with anchors iii) tenotomy of the long head of the biceps tendon iv) tenodesis of the long head of the biceps tendon or v) combination of the above. All patients were preoperatively informed of their shoulder pathology and the possible arthroscopic treatments. In every case, preoperatively physical examination by the same surgeon as well as magnetic resonance imaging were performed. Speed and O´Brien tests were positive in most cases during the clinical examination. Due to the anatomic relation of the long head of biceps tendon and the rotator cuff, the examination tests are not 100% specific. All shoulder arthroscopies were performed in lateral decubitus position. The definitive diagnosis and classification of the SLAP lesion were made during the arthroscopy.

## 4 RESULTS

According to our experience of the clinical outcome after surgical therapy of the SLAP lesion, an algorithm regarding the treatment choices was created. The arthroscopic treatment of symptomatic SLAP lesions in young active patients leads to excellent clinical results with reduced pain and return to previous daily activity or sport level. The appropriate treatment is related to many factors, such as: 1. the type of the lesion, 2. the age of the patient, 3. the gender of the patient, 4. the cause of the lesion (traumatic or non-traumatic), 5. the functional requirement of the patient, 6. the level of sporting activity, and 7. the preoperative expectations of the patient as far as the aesthetic outcome is concerned. Many different surgical approaches exist regarding the fixation of a SLAP lesion, as far as the number of the suture anchors and their location is concerned.

The most common variant of SLAP lesions is type II. Interestingly, the treatment of this type rises most controversies among shoulder surgeons. For a type III SLAP lesion, it is generally suggested to excise the bucket-handle tear of the labrum. For the type IV SLAP lesion is also commonly accepted to perform a tenotomy or tenodesis of the long head of biceps tendon. The detached part of the labrum is most of the times removed and not reattached to the glenoid. In young active patients with good quality of labrum, a reattachment of the labrum to the glenoid simultaneously with a tenodesis/tenotomy of the long head of biceps tendon could also be performed.

Thus, our suggestion for the arthroscopic treatment of SLAP lesion is summarized in the algorithm below.

## 5 DISCUSSION

In the literature, the clinical outcome after surgical treatment of SLAP lesions has been extensively discussed. Beyzadeoglu *et al*. [5] described the most common pathologies coexisting with a SLAP lesion in elite athletes. These include the Bankart lesion and partial cuff tear. In their study an anatomic repair of the SLAP lesion as well as an aggressive rehabilitation for an elite athlete to reach the previous level of athletic performance was suggested.

The initial treatment of a patient with a SLAP lesion should be conservative. Some studies demonstrate that non-operative treatment can be successful in many cases [9]. Surgical treatment of SLAP lesions in middle-aged and older patients is controversial. Many surgeons prefer the tenotomy of the long head of biceps in elderly patients as first treatment choice. The tenotomy of the biceps tendon is favoured when other entities, such as rotator cuff tears, coexist [10]. Boileau *et al*. [11] argue that tenodesis of the biceps tendon in overhead athletes has better clinical results than SLAP repair. Isolated type II SLAP lesion also seems to have a better clinical outcome after a biceps tenodesis compared with SLAP repair in patients older than 35 years [12]. However, Ek *et al*. [13] suggest that in younger patients (<35 years) with healthy-looking labral tissue a SLAP repair should be performed. On the other hand, in patients older than 35 years with lower activity level and less functional requirement a tenodesis of the biceps tendon is the treatment of choice. Over the last 10 years the number of SLAP repairs seems to decrease while the number of the biceps tenodesis has increased, as described in a study of Erickson *et al*. [14]. The average age of the patients, who underwent SLAP repair has also decreased.

According to our opinion, anti-inflammatory drugs, physiotherapy, change or improvement of sport technique should be initially suggested as a treatment option. Possible deficiencies in the kinetic chain of skapulothoracic joint should be also diagnosed and appropriately treated simultaneously. The initial conservative approach should last at least 3-6 months. If the patient still complains about joint symptoms, which prevent daily living or sport activities, then an operative treatment should be discussed. The advantages and disadvantages of the treatment (labrum fixation, tenotomy or tenodesis of the biceps tendon) should always be extensively discussed with the patient preoperatively.

In SLAP lesion type I we recommend only an arthroscopic debridement of the superior labrum.

For SLAP lesions type II we recommend in young and active patients with a recent trauma a SLAP fixation with one or two suture anchors. Especially when the cause of the lesion is traumatic, the superior labrum should be fixated to the glenoid. During the arthroscopy, we use the standard posterior portal as viewing portal. The stability of the SLAP complex is tested with a probe from the standard anterior portal. If a suture anchor is needed, we use the anterolateral portal for the anchor placement. In most cases the posterior part of the SLAP is also detached. For this reason, we try to implant the anchor to the posterior part of the superior glenoid. In our department, we perform a SLAP repair only in young (<40 years) and active patients, with an obvious and recent trauma, a good quality of labral tissue and a clearly detached labrum from the glenoid. In cases where the patients are older than 40 years old, having low sport activity level or in cases with co-existed intraarticular pathologies, we recommend a tenotomy or tenodesis of the long head of biceps tendon. In middle-aged and thin patients, we prefer to perform a tenodesis rather than a tenotony, due to the better cosmetic outcome. Because of the prolonged postoperative pain or the mild stiffness of the joint, we tend to perform in type II SLAP lesions more often tenotomy or tenodesis than fixation in the glenoid.

In cases of the type III SLAP lesions, we recommend a resection of the bucket-handle tear of the superior labrum. The resection is in most cases sufficient, the rest of the labrum is stable und no reattachment to the glenoid is necessary.

For the type IV SLAP lesions, we suggest a tenotomy or a tenodesis of the long head of the biceps tendon regardless to the other criteria, such as patient’s age or activity level. We prefer to perform the tenodesis using a suture anchor in the bicipital groove. In case of a SLAP tear revision surgery we recommend a biceps tenodesis and prefer the sub-pectoral type of tenodesis with one anchor.

## CONCLUSION

The evaluation and treatment of SLAP lesions continue to be controversial [15]. The most common challenge for both the shoulder surgeon and the patient is how to treat the biceps pathology. Many surgeons prefer to preserve the biceps tendon. On the other hand, many other prefer to perform a tenotomy and keep the biceps tendon extra-articularly. In this article, an algorithm is presented on how to treat a SLAP lesion according to the type of lesion, age, gender, functional demands and sport activity level of the patient.

## Figures and Tables

**Fig. (1):**
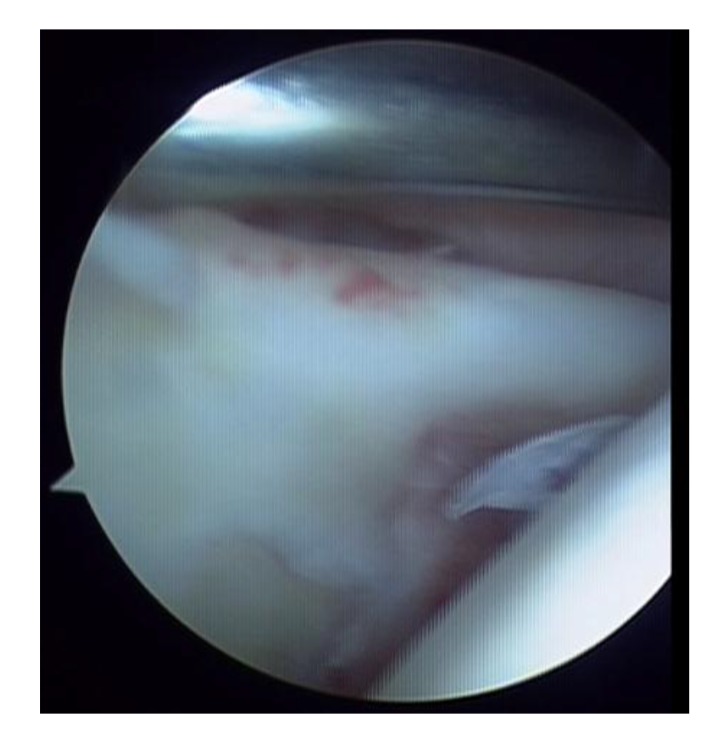
SLAP I lesion.

**Fig. (2):**
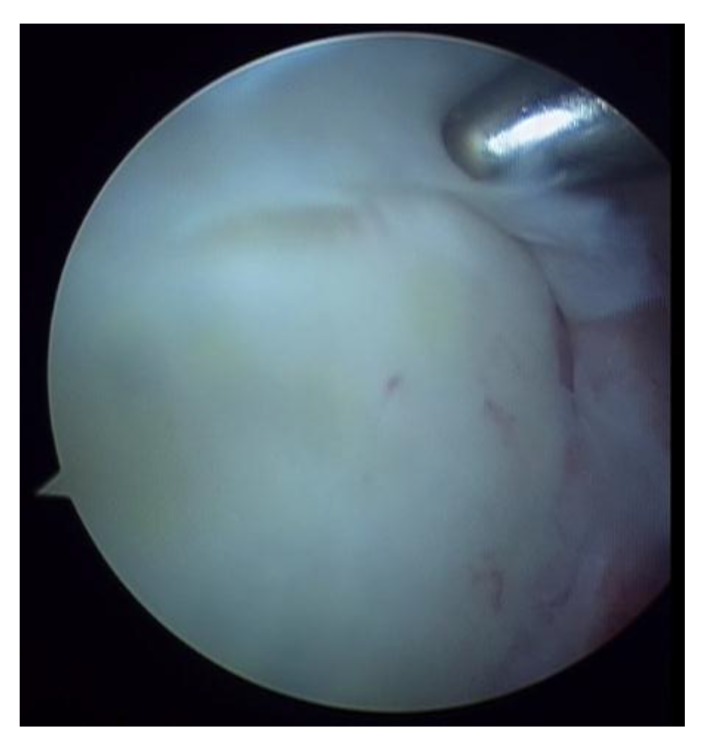
SLAP II lesion.

**Fig. (3):**
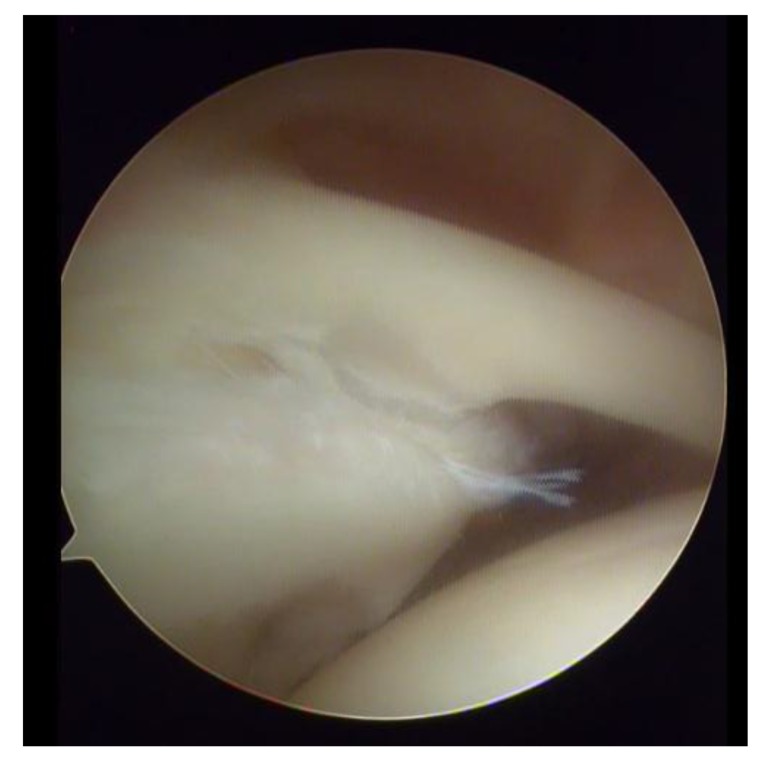
SLAP III lesion.

**Fig. (4):**
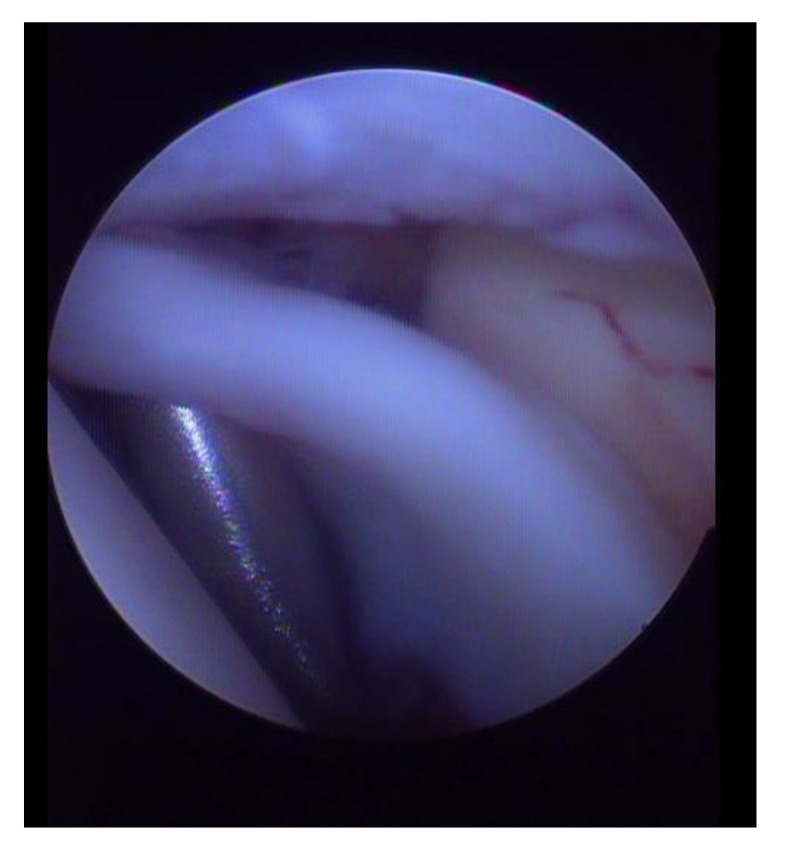
SLAP IV lesion.

**Fig. (5):**
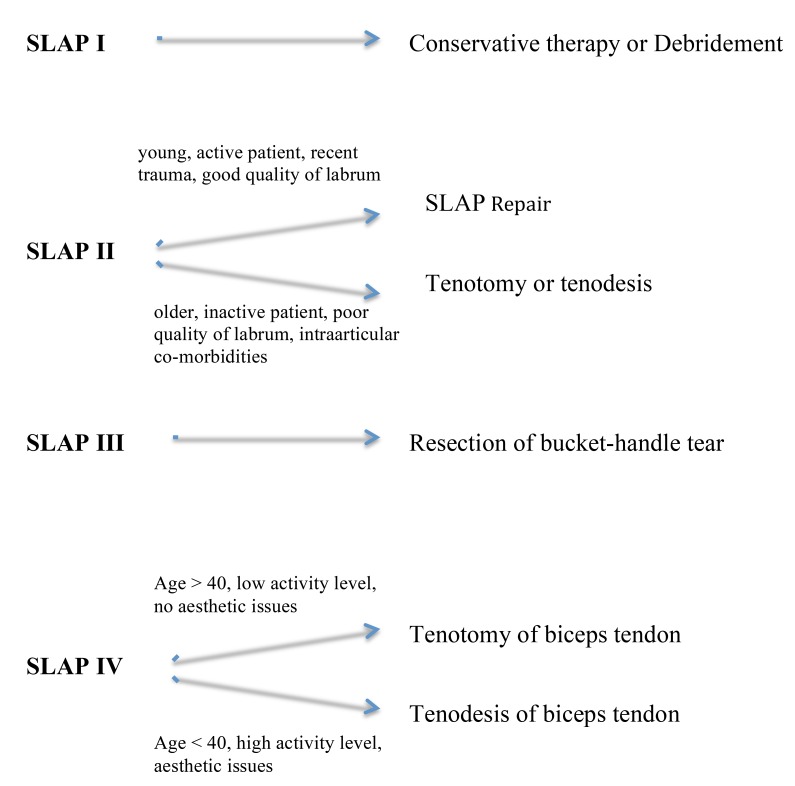
Treatment algorithm for SLAP lesions.

## LIST OF ABBREVIATIONS

BMI: Body Mass Index
MRA: Magnetic Resonance Arthrography
SLAP: Superior Labrum from Anterior to Posterior

## References

[1] Ahsan Z.S., Hsu J.E., Gee A.O. (2016). The Snyder classification of superior labrum anterior and posterior (SLAP) lesions.. Clin. Orthop. Relat. Res..

[2] Andrews J.R., Carson W.G., McLeod W.D. (1985). Glenoid labrum tears related to the long head of the biceps.. Am. J. Sports Med..

[3] Snyder S.J., Karzel R.P., Del Pizzo W., Ferkel R.D., Friedman M.J. (1990). SLAP lesions of the shoulder.. Arthroscopy.

[4] Aydin N., Sirin E., Arya A. (2014). Superior labrum anterior to posterior lesions of the shoulder: Diagnosis and arthroscopic management.. World J. Orthop..

[5] Beyzadeoglu T., Circi E. (2015). Superior labrum anterior posterior lesions and associated injuries..

[6] McCormick F., Bhatia S., Chalmers P., Gupta A., Verma N., Romeo A.A. (2014). The management of type II superior labral anterior to posterior injuries.. Orthop. Clin. North Am..

[7] Huri G., Hyun Y.S., Garbis N.G., McFarland E.G. (2014). Treatment of superior labrum anterior posterior lesions: A literature review.. Acta Orthop. Traumatol. Turc..

[8] Jee W.H., McCauley T.R., Katz L.D., Matheny J.M., Ruwe P.A., Daigneault J.P. (2001). Superior labral anterior posterior (SLAP) lesions of the glenoid labrum: Reliability and accuracy of MR arthrography for diagnosis.. Radiology.

[9] Popp D., Schöffl V. (2015). Superior labral anterior posterior lesions of the shoulder: Current diagnostic and therapeutic standards.. World J. Orthop..

[10] Brockmeyer M., Tompkins M., Kohn D.M., Lorbach O. (2016). SLAP lesions: A treatment algorithm.. Knee Surg. Sports Traumatol. Arthrosc..

[11] Boileau P., Parratte S., Chuinard C., Roussanne Y., Shia D., Bicknell R. (2009). Arthroscopic treatment of isolated type II SLAP lesions: Biceps tenodesis as an alternative to reinsertion.. Am. J. Sports Med..

[12] Denard P.J., Lädermann A., Parsley B.K., Burkhart S.S. (2014). Arthroscopic biceps tenodesis compared with repair of isolated type II SLAP lesions in patients older than 35 years.. Orthopedics.

[13] Ek E.T., Shi L.L., Tompson J.D., Freehill M.T., Warner J.J. (2014). Surgical treatment of isolated type II superior labrum anterior-posterior (SLAP) lesions: Repair versus biceps tenodesis.. J. Shoulder Elbow Surg..

[14] Erickson B.J., Jain A., Abrams G.D., Nicholson G.P., Cole B.J., Romeo A.A., Verma N.N. (2016). SLAP lesions: Trends in treatment.. Arthroscopy.

[15] Kibler W.B., Sciascia A. (2016). Current practice for the surgical treatment of SLAP lesions: A systematic review.. Arthroscopy.

